# Case report: Early (molecular) diagnosis is the clue: report on ALDH7A1 deficiency in newborns

**DOI:** 10.3389/fgene.2024.1464556

**Published:** 2024-09-12

**Authors:** Patryk Lipiński, Katarzyna Wójcicka-Kowalczyk, Anna Bogdańska, Ewa Ehmke, Magdalena Pajdowska, Katarzyna Skrzypek, Agnieszka Charzewska, Dorota Hoffman-Zacharska

**Affiliations:** ^1^ Institute of Clinical Sciences, Maria Skłodowska-Curie Medical Academy, Warsaw, Poland; ^2^ Department of Pediatrics, Bielański Hospital, Warsaw, Poland; ^3^ Department of Neonatology and Neonatal Intensive Care, The Children’s Memorial Health Institute, Warsaw, Poland; ^4^ Department of Clinical Biochemistry, The Children’s Memorial Health Institute, Warsaw, Poland; ^5^ Department of Pediatrics, Nutrition and Metabolic Diseases, The Children’s Memorial Health Institute, Warsaw, Poland; ^6^ Masdiag-Diagnostic Mass Spectrometry Laboratory, Warsaw, Poland; ^7^ Department of Medical Genetics, Institute of Mother and Child, Warsaw, Poland

**Keywords:** developmental and epileptic encephalopathies, pyridoxine-dependent epilepsy, 6-oxopipecolate, next-generation sequencing, *ALDH7A1* gene

## Abstract

The first-tier genetic testing for developmental and epileptic encephalopathies (DEE) is now increasingly used in routine clinical practice. Antiquitin deficiency, also referred to as pyridoxine-dependent epilepsy (PDE-ALDH7A1), represents an inherited metabolic disorder with the phenotype of an early infantile DEE. In addition to the fact that biochemical biomarkers of PDE-ALDH7A1, including α-aminoadipic semialdehyde dehydrogenase, pipecolic acid (PA), Δ1-piperideine-6-carboxylate, and 6-oxopipecolate (6-oxo-PIP), are well-characterized, and their analysis and usefulness have some limitations. Here, we describe the case of a newborn presenting with seizures from the first hours of life, who was resistant to standard antiepileptic drugs and was found to be a biallelic compound heterozygote of two clearly pathogenic variants in the *ALDH7A1* gene based on targeted next-generation sequencing (NGS). The diagnostic process of PDE-ALDH7A1 was limited by the possibility to determine only urinary PA and 6-oxo-PIP (urinary organic acid profile using the GC–MS method), and the exogenous peak of levetiracetam, due to the fact that it has a similar retention time as 6-oxo-PIP, masked the detection of 6-oxo-PIP.

## 1 Introduction

Pyridoxine-dependent epilepsy (PDE-ALDH7A1), also referred to as early-onset vitamin B6-dependent epilespy-4 (EPEO4, OMIM 266100) due to the deficiency of α-aminoadipic semialdehyde dehydrogenase (α-AASA, also known as antiquitin; E.C. 1.2.1.3), is a developmental and epileptic encephalopathy (DEE) characterized by seizures resistant to standard medications and responsive to pyridoxine treatment (Coughlin et al., 2022; Coughlin et al., 2021; Coughlin et al., 2019; Stockler et al., 2011). It is caused by homozygous or compound heterozygous mutations in the *ALDH7A1* gene (OMIM 107323) (Coughlin et al., 2022; Coughlin et al., 2021; Coughlin et al., 2019; Stockler et al., 2011). The *ALDH7A1* gene is localized in *locus* 5q32.2 and consists of 18 exons encoding 539-amino acid protein—aldehyde dehydrogenase. So far, over 200 pathogenic variants in this gene have been identified (HGMD Professional 2024.1, https://my.qiagendigitalinsights.com/bbp/view/hgmd/pro/all.php, accession 30.05.2024), with the most frequently reported *p*. Glu427GLn missense variant (Coughlin et al., 2019).

Antiquitin deficiency results in the accumulation of pipecolic acid, Δ1-piperideine-6-carboxylate (Δ1-P6C), α-AASA, and 6-oxopipecolate (6OP, 6-oxo-PIP) in body fluids, usually measured in urine, plasma, and cerebrospinal fluid (CSF) (Coughlin et al., 2022; Coughlin et al., 2021; Coughlin et al., 2019; Stockler et al., 2011; Kuhara et al., 2020). Lysine reduction therapies, including a lysine-restricted diet and L-arginine therapy in addition to pyridoxine, reduce the accumulation of the abovementioned putative neurotoxic metabolites, aiming to provide better seizure control and cognitive outcome (Coughlin et al., 2022; Coughlin et al., 2021; Coughlin et al., 2019; Stockler et al., 2011; Kuhara et al., 2020). Timely confirmation of PDE-ALDH7A1 and disease-specific treatment implementation is thus essential. The diagnosis is made on the basis of detection of biomarkers and/or by genetic testing. The latter is now increasingly accepted as the first-tier testing for epileptic encephalopathies, even when pathognomonic biomarkers exist (Coughlin et al., 2022; Coughlin et al., 2021; Coughlin et al., 2019; Stockler et al., 2011; Kuhara et al., 2020).

The aim of this paper was to provide a case report of a newborn with PDE-ALDH7A1, highlighting the limitations of biomarkers usefulness in favor of next-generation sequencing (NGS) technology application in clinical practice.

## 2 Case report

The patient (boy) was the first child of non-consanguineous Polish parents born from an uneventful pregnancy at 40 weeks of gestation with a birth mass of 4.290 g; a head circumference of 38 cm; and Apgar scores of 8, 9, and 9 at 1, 5, and 10 min, respectively. Due to seizures on postnatal day 1 with a generalized paroxysmal activity demonstrated in EEG, phenobarbital treatment was implemented. Levetiracetam was subsequently added in the following days due to getting no response. Additionally, 200 mg of intravenous (IV) pyridoxine was daily administered for several days. The seizures were finally brought under control with the normalization of EEG activity. Both phenobarbital and levetiracetam were then continued. Biochemical results, including plasma lactate, uric acid, ceruloplasmin, and ammonia, as well as urinary organic acid profile, plasma amino acids, acylcarnitine profile, transferrin isoelectric focusing, very long-chain fatty acids, and CSF glucose, lactate, amino acids, and neurotransmitters, were unremarkable. Brain magnetic resonance (MR) imaging at the 10^th^ day of life showed non-specific bilaterally symmetrical hyperintense changes on T2-weighted imaging. The NGS panel of genes related to epileptic encephalopathies was launched.

From the 20^th^ day of life, generalized tonic–clonic seizures with a burst-suppression pattern in EEG were observed. The patient was transferred to the reference center (CMHI). Oral pyridoxine (30 mg/kg/day) was re-administered, while phenobarbital and levetiracetam were provided in maximal therapeutic doses. Biochemical screening into inborn errors of metabolism (IEM) revealed no abnormalities; however, it was a difficulty in the assessment of urinary 6-oxopipecolic acid by gas chromatography–mass spectrometry (GC/MS) analysis due to the exogenous peak from levetiracetam (masking the 6-oxo-PIP peak). Brain MR demonstrated the same changes, as noted previously. Within 3 days of treatment, the seizures were under control together with the cessation of epileptiform activity in EEG.

The patient underwent a targeted NGS genetic test that included a panel of 83 genes associated with the occurrence of neonatal seizures and epileptic encephalopathies (NBEv.1/2018, https://zgm.imid.med.pl/panele-ngs/). The pathogenicity of the identified variants was assessed based on classification data according to ACMG (Richards et al., 2015), the human pathogenic mutation databases HGMD Professional 2023.3 (https://my.qiagendigitalinsights.com/bbp/view/hgmd/pro), and ClinVar (https://www.ncbi.nlm.nih.gov/clinvar/). The variants identified in this study were described according to HGVS nomenclature recommendations (http://varnomen.hgvs.org/recommendations).

**FIGURE 1:**
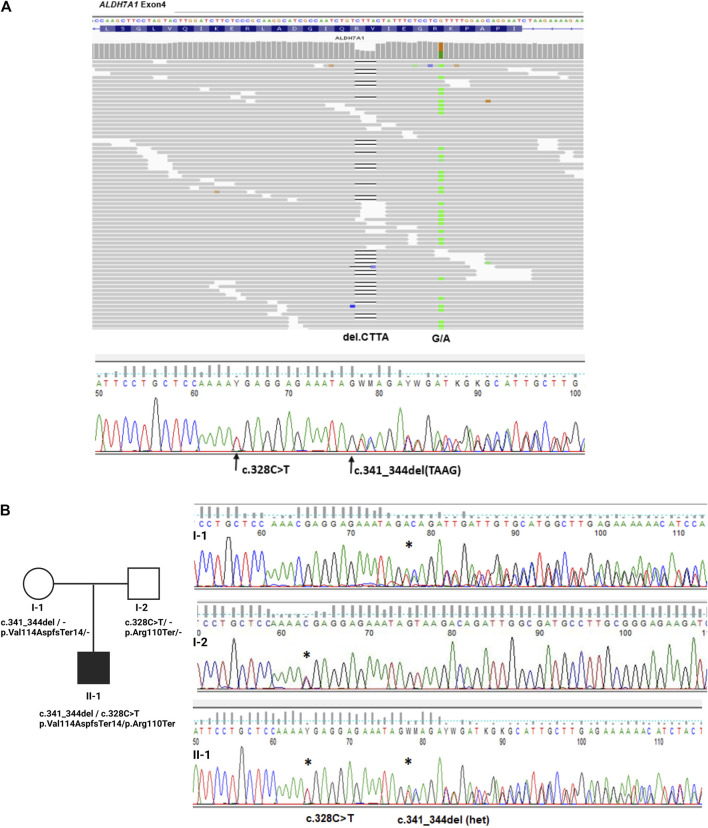
Genetic examination and family study of variants in the *ALDH7A1* gene. **(A)**. Integrative genomics viewer (IGV) visualization of read alignments in the region of *ALDH7A1* exon 4 with marked identified two variants in the proband and Sanger sequencing results confirming NGS’ finding. **(B)**. Analysis of the inheritance of *ALDH7A1* variants in proband (II–1) and hid parents (I–1, I–2), confirming the biallelic localization of identified variants in exon 4 of the gene.

The NGS results showed the presence of two heterozygous truncating variants in the *ALDH7A1* gene, frameshift *p*. Val114AspfsTer14 (c.341_344del) and nonsense *p*. Arg110Ter (c.328C>T), see Figure 1A. The segregation analysis of the *ALDH7A1* gene variants in the proband’s family, by direct Sanger sequencing of the gene’s regions carrying identified mutations, indicated that both variants were inherited; see Figure 1B. Both parents were carriers, which confirmed biallelic localization of identified variants. The proband exhibited a compound heterozygous configuration variant (genotype, *ALDH7A1* (NM_001182.5, c. [341_344del];[c.328C>T]; *p* [(Val114AspfsTer14)]; [(Arg110Ter)]).

The final diagnosis of PDE-ALDH7A1 was established; a lysine-restricted diet (based on the additional supply of GA1 Anamix Junior) with the addition of arginine was then initiated. During the first year of life, the patient remained seizure-free on oral pyridoxine (30 mg/kg/day) as monotherapy. At the last follow-up (age of 16 months), the patient presented with normal psychomotor development, while the urinary GC/MS analysis demonstrated the trace of 6-oxopipecolate and elevated 4-pirydoxinic acid (secondary to treatment).

## 3 Discussion

### 3.1 Clinical difficulties/pitfalls

PDE-ALDH7A1, along with molybdenum cofactor deficiency, isolated sulfite oxidase deficiency, neonatal non-ketotic hyperglycinemia, congenital disorders of glycosylation, Zellweger syndrome, adenylosuccinate lyase deficiency, and congenital hypophosphatasia, represents the group of inherited metabolic disorders (IMDs), presenting as an early-infantile developmental and epileptic encephalopathy (EIDEE) (Zuberi et al., 2022). Most PDE-ALDH7A1 (classic type) patients develop seizures within first hours to days of life or even antenatally (excessive fetal movements). A variety of seizure types could be observed, including focal seizures, spasms, and generalized tonic–clonic seizures (Coughlin et al., 2022; Coughlin et al., 2021; Coughlin et al., 2019; Stockler et al., 2011; Kuhara et al., 2020). EEG in neonates can show a burst-suppression pattern, however, focal or multifocal discharges may be seen against a background of slow rhythms (Coughlin et al., 2022; Coughlin et al., 2021; Coughlin et al., 2019; Stockler et al., 2011; Kuhara et al., 2020). PDE-ALDH7A1 should be considered in all infants with drug-resistant seizures; however, some children may be partially responsive to anti-seizure medications (Kaminiów et al., 2021). The diagnostic and therapeutic procedure is administering 100 mg of pyridoxine intravenously; in individuals with PDE, clinical seizures cease over a period of several minutes, and if a clinical response is not demonstrated, the dose should be repeated up to a maximum of 500 mg (Kaminiów et al., 2021). PDE could be also clinically proven by withdrawing pyridoxine from a patient who had been successfully treated, in order to demonstrate the recurrence of seizures (generally within a few days) and then be controlled once again by reintroducing pyridoxine. The interval to the recurrence of seizures usually lasts a few days; however, it is longer in the newborn period, ranging even up to 18 days (Coughlin et al., 2022; Coughlin et al., 2021; Coughlin et al., 2019; Stockler et al., 2011; Kuhara et al., 2020).

The paper described a case of newborn with seizures observed from first hours of the patient’s life and resistant to two anti-epileptic drugs. The results of standard biochemical analyses as a screening of inherited metabolic disorders were unremarkable. Pyridoxine was administered (with a positive clinical response) and discontinued after several days with the recurrence of seizures. Therefore, we would like to highlight that all neonatologists must be alert to a timely and appropriate pyridoxine implementation in PDE suspicion.

### 3.2 Limitations of diagnostic biomarkers

PDE-ALDH7A1 diagnostic biomarkers include α-AASA, Δ1-P6C, PA, and 6-oxo-PIP, which can be measured in urine, blood, and CSF. Ideally, urine and plasma samples should be taken prior to treatment with pyridoxine; however, this should not delay the initiation of therapy.

Despite the unclear role in lysine degradation, PA was the first biomarker used to diagnose patients with PDE-ALDH7A1 (Plecko et al., 2007; Mercimek-Mahmutoglu et al., 2013; Plecko et al., 2005). PA is now regarded as not the best diagnostic biomarker due to several reasons. First of all, its concentration in plasma could be normal in ALD7A1-deficient patients (Mercimek-Mahmutoglu et al., 2013). Second, as plasma PA is also elevated in peroxisomal disorders and liver disease, even its elevated levels will not confirm the diagnosis of PDE-ALDH7A1 (Mercimek-Mahmutoglu et al., 2013). A more marked increase of PA in CSF compared to plasma could help discriminate pyridoxine-dependent epilepsy from other possible defects with elevated PA in plasma (Plecko et al., 2005). Finally, as shown in the study of Plecko et al. (2005), urinary PA had the tendency to decrease and then normalize upon pyridoxine treatment, while plasma and CSF PA remained elevated at all ages even upon pyridoxine treatment. Furthermore, plasmatic PA levels may normalize after many months to years of successful treatment with pyridoxine, giving its role in the treatment monitoring as negligible.

Two other PDE-ALDH7A1 metabolites, namely, α-AASA and Δ1-P6C, are in equilibrium (Coughlin et al., 2021). These two biomarkers are very unstable compounds as they degrade rapidly within a few hours at ambient temperature. Sample handling is critical for reliable results; otherwise, poor sample processing could potentially result in false negative (Sadilkova et al., 2009; Struys et al., 2012). Furthermore, the method is semi-quantitative and only established in a few specialized laboratories worldwide. Finally, patients with molybdenum cofactor deficiency and isolated sulfite oxidase deficiency have been reported with mild elevations of α-AASA/Δ1-P6C due to the secondary inhibition of α-AASA dehydrogenase (Mills et al., 2012).

In 2019, Wempe et al. developed a LC–MS/MS-based method to quantify a novel PDE-ALDH7A1 metabolite, namely, 6-oxo-pipecolate (6-oxo-PIP), being an intermediate metabolite between Δ1-P6C and α-AASA in lysine oxidation (Wempe et al., 2019). Unlike previously identified biomarkers, 6-oxo-PIP was found to be relatively stable at room temperature and could be added to the current newborn screen paradigm (Wempe et al., 2019). However, in the recent study of Khalil et al. (2024), 6-oxo-PIP levels in the urine were found within the normal range in 33% of the patients below 6 months of age, demonstrating that urinary 6-oxo-PIP may not be a suitable biomarker for ALDH7A1 deficiency in neonates.

The patient described here was transferred to the reference center in Warsaw. However, the diagnostic process of PDE-ALDH7A1 was limited by the possibility to determine only urinary PA and 6-oxo-PIP (urinary organic acid profile using the GC–MS method). Furthermore, the exogenous peak of levetiracetam (quite massive in patient’s sample), due to the fact that it has a similar retention time as 6-oxo-PIP, masked the detection of 6-oxo-PIP.

### 3.3 Molecular analyses

Genetic testing, either using the NGS-targeted gene panels (TGP) or whole-exome sequencing (WES) sequencing, is currently a powerful diagnostic tool in clinical practice. Both WES and TGP have increased the knowledge about the genetic background of DEEs, allowing the identification of the most of more than 100 DEEs’ genes [OMIM, DEE PS308350], understanding the pathogenesis of these diseases (Ruggiero et al., 2023). A detailed description of individual cases and bigger cohorts allowed the characterization of new phenotypic subtypes and visualized the complexity of genotype–phenotype associations (Salinas et al., 2021). In addition, the excellent cost–benefit ratio of NGS-based tools has facilitated their incorporation into the diagnostic workup. As a result, molecular analyses are increasingly accepted as the first-tier testing for epileptic encephalopathies.

Despite the diagnostic implication of biomarkers (PA, α-AASA/Δ^1^-P6C, and 2-oxo-PIP), the confirmation of PDE-ALDH7A1 requires the evaluation of the *ALDH7A1* gene, which can be done through specific gene testing, multigene panels, and comprehensive genomic testing. With the usefulness of (emergency/rapid) NGS studies, some patients with PDE-ALDH7A1 were diagnosed prior to the administration of pyridoxine treatment (Coughlin and Gospe, 2023). NGS has also contributed to the field of PDE by unraveling late-onset cases of PDE-ALDH7A1 and cases not responsive to pyridoxine, like pyridoxamine 5′-phosphate oxidase (PNPO) deficiency (OMIM 610100), which should be treated with the implementation of pyridoxal phosphate (PLP) (Falsaperla et al., 2018). In 2016, the whole-exome sequencing study of two children with pyridoxine-dependent epilepsy revealed a novel genetic cause of PDE associated with the homozygous nonsense mutation in the *PROSC* gene encoding a PLP-binding protein.

To finalize the abovementioned concerns regarding a diagnostic utility of both biomarkers and genetic analyses, we would like to emphasize the results of retrospective observational study on children with PDEs, diagnosed and followed up in Italian Pediatric Departments (Falsaperla et al., 2018). A total of 8 out of 16 patients (50%) were found positive for ALDH7A1 deficiency, 3 (18.75%) had a mutation of the *PNPO* gene, 2 (12.5%) had a mutation of the *PROSC* gene, and 3 (18.75%) had negative genetic results. Only 6 (37.5%) patients were diagnosed with elevated serum and/or urine alpha-aminoadipic semialdehyde and/or pipecolic acid, while the rest of the children (62.5%) had an *ex juvantibus* diagnosis. Further research is needed to better characterize both biochemical and genetic aspects of the PDEs.

## Data Availability

The original contributions presented in the study are included in the article/Supplementary Material, further inquiries can be directed to the corresponding author.
